# Study of Cytotoxic and Photodynamic Activities of Dyads Composed of a Zinc Phthalocyanine Appended to an Organotin

**DOI:** 10.3390/ph14050413

**Published:** 2021-04-28

**Authors:** Isabelle Toubia, Christophe Nguyen, Stéphane Diring, Marine Pays, Elodie Mattana, Philippe Arnoux, Céline Frochot, Magali Gary-Bobo, Marwan Kobeissi, Fabrice Odobel

**Affiliations:** 1 CEISAM, Chimie Et Interdisciplinarité, Synthèse, Analyse, Modélisation, CNRS, UMR CNRS 6230, Université de Nantes, 2, rue de la Houssinière—BP 92208, CEDEX 3, 44322 Nantes, France; isabelle.toubia@univ-lorraine.fr (I.T.); Stephane.Diring@univ-nantes.fr (S.D.); 2 LRGP, Laboratoire Réactions et Génie des Procédés, UMR 7274 CNRS-Université de Lorraine, 1 rue Grandville, 54000 Nancy, France; philippe.arnoux@univ-lorraine.fr; 3 IBMM, Université de Montpellier, CNRS, ENSCM, 34093 Montpellier, France; christophe.nguyen@umontpellier.fr (C.N.); paysmarinepro@gmail.com (M.P.); mattana.e@gmail.com (E.M.); 4 Laboratoire Rammal Rammal, Equipe de Synthèse Organique Appliquée SOA, Faculté des Sciences 5, Université Libanaise, Nabatieh, Lebanon

**Keywords:** photodynamic therapy, chemotherapy, zinc (II) phthalocyanine, tin complex

## Abstract

The combination of photodynamic therapy and chemotherapy is a promising strategy to enhance cancer therapeutic efficacy and reduce drug resistance. In this study two zinc(II) phthalocyanine-tin(IV) conjugates linked by a triethylene glycol chain were synthesized and characterized. In these complexes, the zinc(II) phthalocyanine was used as a potential photosensitizer for PDT and the tin complex was selected as cytostatic moiety. The two dyads composed of zinc(II) phthalocyanine and tin complexes exhibited high cytotoxicity, in absence of light stimulation, against MCF-7 human breast cancer cells with low LC_50_ values in the range of 0.016–0.453 µM. In addition, these complexes showed superior cytotoxicity than their mixture of equimolar component, accompanied with a higher activity towards cancer cells compared to human healthy fibroblasts. However, under irradiation of the zinc phthalocyanine unit (at 650 nm) no photodynamic activity could be detected, due to the most likely quenching of zinc(II) phthalocyanine singlet excited state by the nearby tin complex according to a photoinduced electron transfer process. This study demonstrates the potential of heterometallic anticancer chemotherapeutics composed of a zinc phthalocyanine and tin complex, and it highlights that the development of such conjugates requires that the sensitizer preserves its photophysical properties and in particular its singlet oxygen sensitization ability in the conjugate in order to combine the PDT activity with the cytotoxicity of the anticancer drug.

## 1. Introduction

Recently, there has been considerable interest in combining photodynamic therapy (PDT) with a chemotherapeutic anticancer agent [1,2,3,4,5,6]. The combination of different therapeutic approaches that acts on distinct disease pathways has shown several advantages, such as enhanced therapeutic efficacy, reduced side effects, and drug resistance problems. There are generally three approaches to combine PDT and chemotherapy, including consecutive administration of a photosensitizer and an anticancer drug, the use of their covalent and noncovalent conjugates, and co-encapsulation of these agents in a polymeric nanocarrier [7,8]. The combination of a photosensitizer with an appended anticancer drug, cisplatin [9,10], carboplatin [11,12], doxorubicin (DOX) [13,14,15], and organometallic ruthenium(II) complexes [16,17] has been attempted, in order to evaluate the possibility of synergistic anticancer properties between the two entities. Interesting results were obtained showing an enhanced anticancer efficacy at lower drug doses [18] due to the dark chemostatic effect of the drug amplified by the PDT effect generated upon light excitation of the photosensitizer.

Tin organometallic derivatives are important classes of organometallic drugs, as they exhibit a wide spectrum of biological effects and have been widely studied as bactericides, fungicides, acaricides and wood preservatives. In addition, some compounds possess antitumor activity. The cytotoxic activity of these compounds results from the inhibition of macromolecular synthesis, the alteration of energy-producing metabolism in the mitochondria, and the reduction of DNA synthesis. In addition, the cytotoxicity induced also arises from their interaction with the cell membrane which increases the concentration of cytosolic Ca^2+^ and induces apoptosis. Phthalocyanines are promising second-generation photosensitizers for PDT, owing to their suitable photo-physical and photo-chemical properties, such as the strong absorption in the tissue-penetrating red visible region and high efficiency in generating singlet oxygen [19,20,21,22,23].

**Figure 1:**
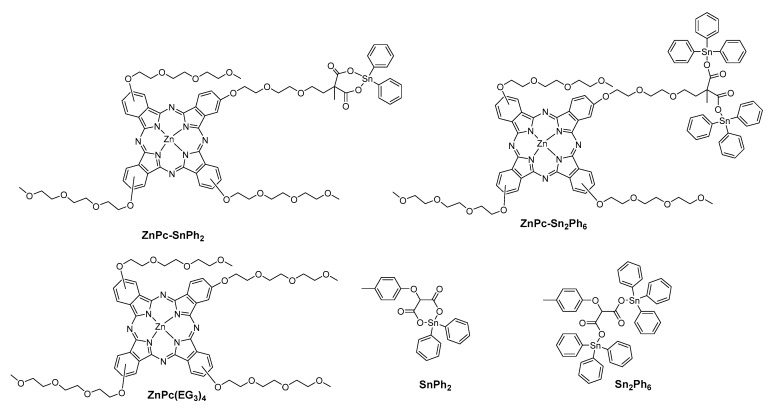
Structures of the compounds investigated in this study.

In this work, we have investigated the combination of tin complexes with a zinc phthalocyanine photosensitizer in order to take advantage of the intrinsic cytotoxicity of tin complex in the dark added to the potentially high phototoxicity of zinc phthalocyanine upon light excitation. The two entities were linked with a triethylene glycol chain, which can enhance the biocompatibility of the system (Figure 1). Although the length of this spacer might have an impact on the biological activity, this parameter was not explored in this study. Two dyads are composed of tetrasubstituted zinc phthalocyanine connected to a tin phenyl complex liganded *via* a malonate anchor. One tin organometallic compound bears two phenyl groups in its coordination sphere (SnPh_2_), while the other is bound to three phenyls (SnPh_3_). Phenyl was selected as organometallic moiety around tin(IV), because it was shown in previous studies that this type of complex gives the most potent anticancer activity [24]. The cytotoxicity of the three dyads was assessed on human healthy (fibroblasts) and cancer cell lines (MCF-7) to quantify their therapeutic potential. It was shown that the two dyads **ZnPc-SnPh_2_** and **ZnPc-Sn_2_Ph_6_** exhibit superior cytotoxicity than their equimolar mixtures of individual components and they display higher selectivity towards MCF-7 carcinoma cell line than healthy cells. On the other hand, these two dyads showed no photodynamic activity under light irradiation.

## 2. Results and Discussion.

### 2.1. Synthesis of the Compounds

**Scheme 1:**
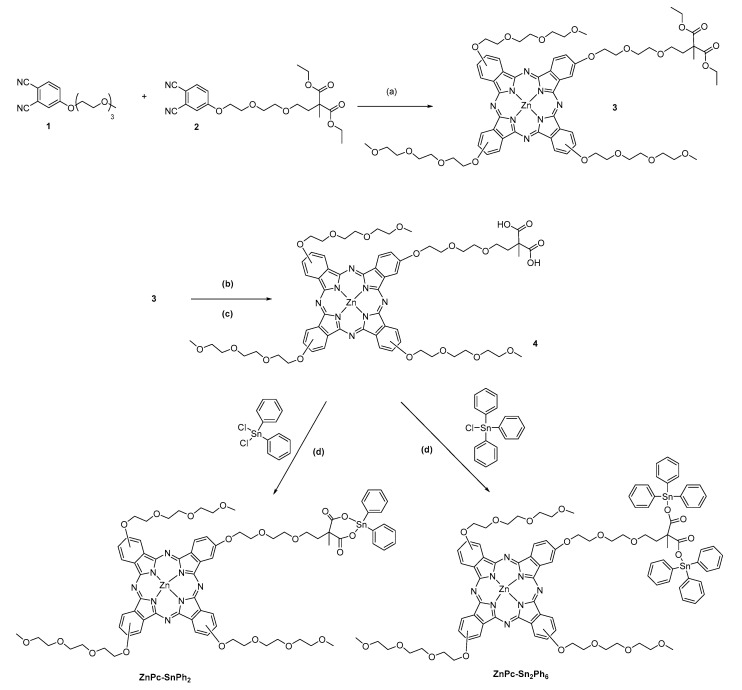
Synthetic route for the preparation of the dyads composed of a zinc phthalocyanine linked to a tin complex. Reagents and conditions: (**a**) n-pentanol, DBU, Zn(OAc)_2_.2H_2_O, 140–150 °C, 12 h, 18%; (**b**) acetone, NaOH, reflux, 2 h; (**c**) HCl, 85%; (**d**) THF, TEA, room temperature, 12 h, 100%.

Scheme 1 illustrates the synthetic route used to prepare the two dyads **ZnPc-SnPh_2_** and **ZnPc-Sn_2_Ph_6_**. An excess of phthalonitrile **1** underwent mixed cyclisation with phthalonitrile **2**^7^ in the presence of Zn(OAc)_2_.2H_2_O and 1,8-diazabicyclo [5.4.0]undec-7-ene (DBU) in n-pentanol to give the «3 + 1» unsymmetrical phthalocyanine **3** in 18% yield (Scheme 1). The ethyl esters of the malonate were subsequently hydrolyzed with sodium hydroxide in acetone and then acidified to afford the phthalocyanine **4** with 85% yield. The introduction of tin complex was accomplished by activating the carboxylic acid group by deprotonation with triethylamine (TEA) before being reacted with the tin precursor containing two or one chloro ligands [24].

The complexes were characterized by proton NMR and elemental analysis as well as by high resolution mass spectrometry. The dyads **ZnPc-SnPh_2_**, **ZnPc-Sn_2_Ph_6_** are soluble in CHCl_3_, DMSO, THF and DMF.

### 2.2. UV-Vis Electronic Absorption Properties

**Figure 2:**
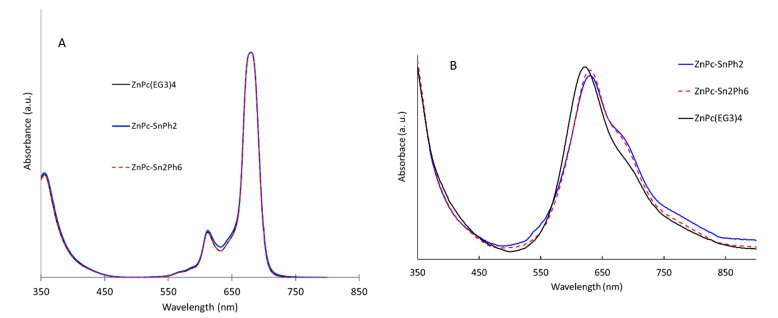
(**A**) Normalized absorption spectra of the phthalocyanine derivatives recorded in chloroform and (**B**) in water with 1% of DMSO at a concentration of about 10^−6^ M.

The absorption spectra of the dyads are compared with those of their components, which are displayed in Figure 2 and the spectroscopic data are gathered in Table 1. In chloroform, **ZnPc(EG_3_)_4_** and the dyads **ZnPc-SnPh_2_** and **ZnPc-Sn_2_Ph_6_** give typical UV-Vis spectra of non-aggregated phthalocyanines showing intense and sharp Q-bands in the red visible region around 680 nm along with the less intense vibronic overtone at 616 nm (Figure 2A). They also displayed a Soret band at 360 nm. The presence of the appended tin complex does not alter the spectrum of the dyads because: (i) it does not absorb beyond 300 nm (Supplementary Figure S1) and (ii) there is no electronic communication between the malonate group and the zinc phthalocyanine on the ground state.

The spectra were also recorded in water with 1% of DMSO to approach the conditions used in biological tests (Figure 2B). The shape and the relative intensity of the Q-bands are clearly different in aqueous environment with a broadening and an inversion of the intensity of the Q(0,0) and Q(0,1) bands, which are located respectively around 620 nm and 690 nm. This change witnesses the formation of H-aggregates. Although the solubility of the compounds is maintained in aqueous medium owing to the ethylene glycol chains, the hydrophobicity of phthalocyanine core induces the formation of π-stacked H-aggregates in aqueous environment.

### 2.3. Infra-Red (IR) Spectroscopic Study

**Figure 3:**
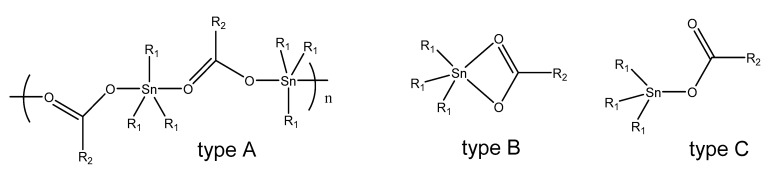
Illustration of three main binding modes of carboxylate ligand with tin(IV).

Carboxylate ligand can chelate tin(IV) cation according to several potential binding modes (Figure 3) [25,26,27]. In solid state, the tin(IV) complexes are usually polymeric compounds with cis-trigonal bipyramidal geometry having five-coordinate tin sites under the bridging mode (type A).

Particularly in solution, the complexes can exist as monomers either under a five-coordinate chelating bridged mode (type B) or as four-coordinated tin with carboxylate acting as a monodentate ligand (type C). ATR-IR spectra can be used as tools to diagnose the mode of coordination of tin complexes [28]. More specifically, the mode of binding can be deduced by the wavenumber difference (ν = ν_as_ − ν_s_) between the carboxylate antisymmetric (ν_as_) and symmetric (ν_s_) stretches. If this difference is larger than 250 cm^−1^, tin(IV) is involved in a tetrahedral geometry (type C binding mode), while values between 250 and 150 cm^−1^ indicate a bridging polymeric structure (type A). When ν is lower than 150 cm^−1^, then carboxylate is bidentate and binds to Sn(IV) under the chelating bridged mode (type B). The strong asymmetric stretching band of the carbonyl group in the acid form of compound **4**, situated at 1725 cm^−1^ has shrunk and is shifted to lower energy (≈1550 cm^−1^) after the reaction with chloro-phenyl tin derivatives, which is a clear indication that complexation of tin by the carboxylate has occurred. The wavenumber values of the stretching bands of the carboxylate before and after complexation of tin(IV) are gathered in Table 2.

For the dyads **ZnPc-SnPh_2_** and **ZnPc-Sn_2_Ph_6_**, the ν values are less than 150 cm^−1^, which indicates that the carboxylate ligand is bidentate and binds to Sn(IV) under the chelating bridged mode.

### 2.4. Fluorescence Spectroscopy

**Figure 4:**
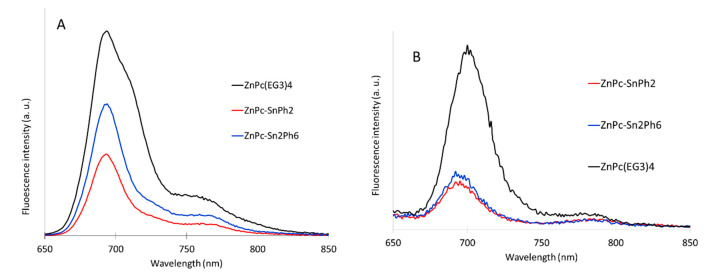
Fluorescence spectra of the dyads **ZnPc-SnPh_2_**, **ZnPc-Sn_2_Ph_6_** recorded in (**A**) CHCl_3_ or (**B**) in water with 1% of DMSO and compared to the spectra of the reference phthalocyanine **ZnPc(EG_3_)_4_** all excited at the same wavelength (λ_exc_ = 615 nm) with an iso-absorbing solution.

Fluorescence spectra of the dyads **ZnPc-SnPh_2_**, **ZnPc-Sn_2_Ph_6_** were compared to those of the reference zinc phthalocyanine **ZnPc(EG_3_)_4_** in order to study the possibility of a singlet excited state quenching by the tin complex. As for the electronic absorption spectra, the fluorescence emission measurements were conducted both in CHCl_3_ and water with 1% of DMSO (Figure 4). Whatever the solvent conditions, the fluorescence intensity of the dyads **ZnPc-SnPh_2_**, **ZnPc-Sn_2_Ph_6_** decreased compare to the reference phthalocyanine **ZnPc(EG_3_)_4_**.

Based on the relative fluorescence intensities, the quenching of **ZnPc-SnPh_2_** and **ZnPc-Sn_2_Ph_6_** is estimated at 60% and 36% in CHCl_3_ and 70% and 75% in water + 1% DMSO respectively. In aqueous medium, the fluorescence intensity is lower than in CHCl_3_, most probably because the H-aggregates impart a supplementary quenching process. In addition to the formation of H-aggregates, another fluorescence quenching process in the dyads could be attributed to photoinduced electron transfer to the tin complex (formation of «ZnP^+^-Sn^III^»). Indeed, energy transfer is unlikely from thermodynamic point of view because the organo-tin complex absorbs in the deep UV, therefore its lowest energy excited state is necessarily higher than that of the singlet excited state of the zinc phthalocyanine (Supplementary Figure S1).

Overall, the fluorescence of the zinc phthalocyanine in these dyads is significantly quenched relative to that of the reference **ZnPc(EG_3_)_4_**. The fluorescence inhibition is due to the presence of tin complex and the formation of H-aggregates, particularly in aqueous environment for the latter. The singlet excited state quenching could affect the efficiency of PDT, due to the reduction of the quantum yield of triplet excited state formation by the intervention of a competitive deactivation processes, which depopulate the singlet excited state (see below).

### 2.5. Singlet Oxygen Measurements

In order to directly estimate the impact of the tin complex on the production of singlet oxygen, the singlet oxygen quantum yield of the compounds was determined in CHCl_3_ and deuterated water + 1% of DMSO by recording the fluorescence intensity of singlet oxygen emission at 1270 nm. D_2_O was chosen instead of H_2_O since the singlet oxygen lifetime value in D_2_O is higher than in H_2_O. This is known that solvents with high vibrational frequencies are more able to quench ^1^O_2_ [29]. The data are collected in Table 3.

In CHCl_3_, the singlet oxygen quantum yield measurements reflect, within experimental errors, the degree of fluorescence quenching. Moreover, the singlet oxygen quantum yield of **ZnPc(EG_3_)_4_** is in agreement with previously reported in DMSO [30]. In D_2_O, however, the singlet oxygen emission is importantly reduced and could not be detected because the quantum yields is too low for the sensitivity of this technique. However, we cannot exclude that the singlet oxygen quantum yield of **ZnPc(EG_3_)_4_** is not null in biological medium as the latter is quite active in PDT (see below).

### 2.6. Biological Effect Analysis of ZnPc(EG_3_)_4_:Cytotoxicity, PDT and ROS Production

**Figure 5:**
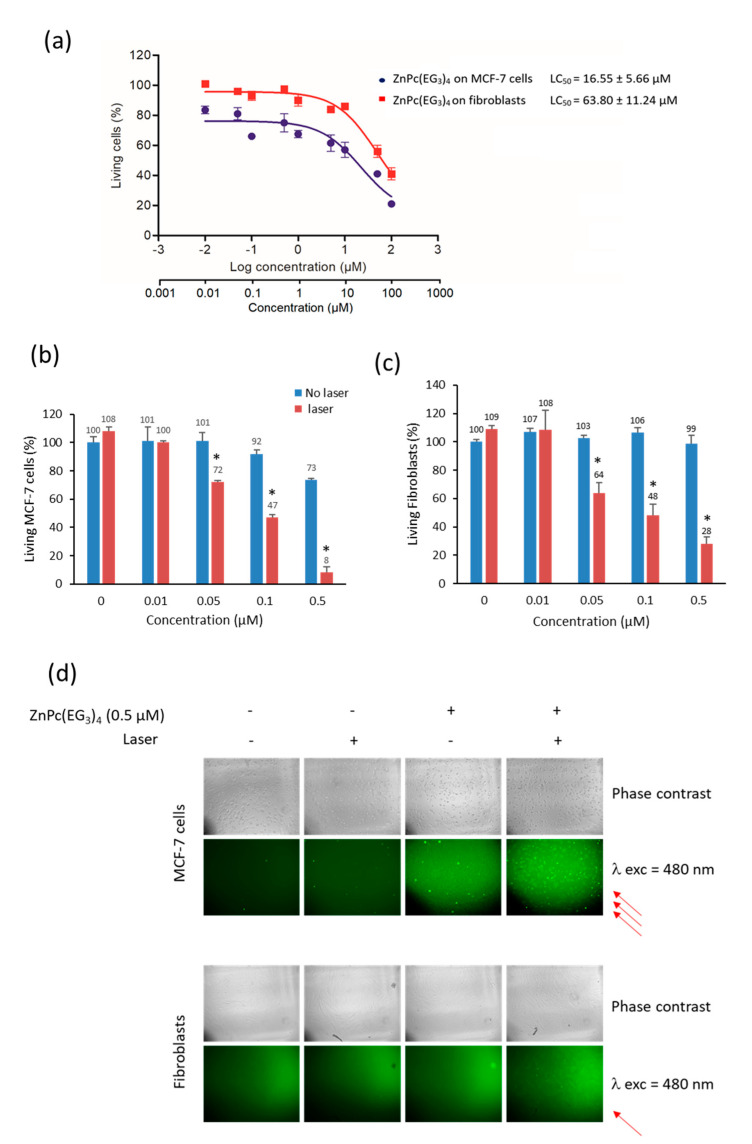
(**a**) Cytotoxicity measurement of increasing concentrations of **ZnPc(EG_3_)_4_**. Dose-responses on cancer MCF-7 cells and healthy fibroblasts incubated 72 h with **ZnPc(EG_3_)_4_**. (**b**) Phototoxicity of the compound **ZnPc(EG_3_)_4_** incubated at increasing concentrations (from 0.01 to 0.50 µM) with cancer cells MCF-7 or (**c**) healthy fibroblasts for 24 h and then irradiated (20 min, λ_exc_ = 650 nm, 39 J.cm^−2^). * *p* < 0.05 statistically different from “No laser”. The bar graph corresponds to the mean ± standard deviation of the values of 2 independent experiments in triplicate. (**d**) Demonstration of ROS production in cells (highlighted by red arrows) incubated for 24 h with 0.50 µM of **ZnPc(EG_3_)_4_**, then incubated with DCFH_2_-DA (20 µM, 45 min) and irradiated for 10 min at 650 nm. The green fluorescence inside the cells reflects ROS production (imaged at 480 nm).

The cytotoxic effect of **ZnPc(EG_3_)_4_** on human breast cancer cells (MCF-7) was determined and compared with that on healthy fibroblasts. For this, a dose-response experiment was performed by incubating cells in the darkness with increasing doses of **ZnPc(EG_3_)_4_** for 72 h. Then, the viability of cells was quantified and reported in Figure 5a. **ZnPc(EG_3_)_4_** exhibited a LC_50_ of 16.55 µM on MCF-7 cells and of 63.80 µM on fibroblasts. This difference (4 fold) between these two cell lines demonstrated that this compound was more cytotoxic on cancer cells than healthy cells.

The photodynamic therapeutic potential was also investigated on breast cancer and healthy cells. Several concentrations of compound (from 0.01 to 0.50 µM) were tested. Cells were incubated 24 h with **ZnPc(EG_3_)_4_** and submitted or not to laser excitation (650 nm, 20 min, 39 J.cm^−2^). Two days later, the viability was quantified MCF-7 cells demonstrated a significant photosensitivity from 0.05 µM of **ZnPc(EG_3_)_4_** (Figure 5b). Indeed, cells submitted to laser excitation exhibited 31% cell death. This PDT effect increased in accordance with increasing concentration of **ZnPc(EG_3_)_4_** to reach 92% cell death when cells were incubated with 0.50 µM of **ZnPc(EG_3_)_4_** and submitted to photoexcitation. This effect is very strong and indicates that the reference compound **ZnPc(EG_3_)_4_** is quite active in PDT, most reasonably upon production of singlet oxygen. We also investigated this PDT efficiency on healthy cells in order to verify if the discriminatory effect already observed on dark cytotoxicity (Figure 5c) could be reproduced under laser excitation. Unfortunately, this effect is about the same on cancer or healthy cells, with a significant effect from 0.05 µM and a high PDT efficiency at 0.50 µM. However, we could note a difference between the two cell lines at this concentration; indeed, only 8% of cancer cells survived when 28% of fibroblasts survived after irradiation.

To identify the phototoxicity mechanism involved here, ROS quantification was performed and reported in Figure 5d. Cells were incubated with DCFH_2_-DA (2′,7′-dichlorodihydrofluorescein diacetate), which is a non-fluorescent molecule. In the presence of ROS this molecule is oxidized into the fluorescent 2′,7′-dichlorodihydrofluorescein (DCF), whose green luminescence can be detected using a fluorescence microscope. The results showed that in the presence of 0.50 µM of **ZnPc(EG_3_)_4_**, light excitation at a wavelength of 650 nm induced green fluorescence inside the cells, mainly on cancer cells but also on healthy cells, thus demonstrating ROS production and confirming cell death induced by PDT effect.

Altogether these data demonstrated that this compound is very efficient for dark cytotoxicity and overall for PDT action, but it is not really discriminant between cancer and healthy cells. Although often, the main limitation of cancer therapy is the poor selectivity of cancer cells inducing a large panel of secondary effects on healthy organs and cells. So we decided to graft organotin complex on **ZnPc(EG_3_)_4_** to improve the effect and the selectivity on cancer cells.

### 2.7. Cytotoxic Potential Analysis of the Dyads ZnPc-SnPh_2_ and ZnPc-Sn_2_Ph_6_ on Cancer and Healthy Cells

In order to determine the therapeutic potential of these new compounds, the cytotoxic activity on MCF-7 cells and also on healthy fibroblasts was studied. Towards this goal, the cells were incubated 72 h in darkness, with increasing concentrations of each compound (from 0.01 to 100 µM). Drug concentrations leading to 50% cell mortality (LC_50_) were determined using the classical sigmoidal dose-response curves of cytotoxicity obtained when plotted as a logarithmic function of the concentration (µM).

**Figure 6:**
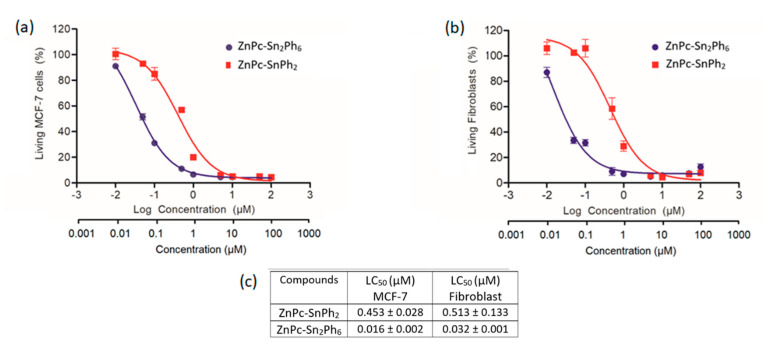
Cytotoxic study of **ZnPc-SnPh_2_** and **ZnPc-Sn_2_Ph_6_**. (**a**) MCF-7 cells and (**b**) healthy fibroblasts were incubated 72 h with increasing concentrations of **ZnPc-SnPh_2_** and **ZnPc-Sn_2_Ph_6_**, and maintained in darkness. (**c**) LC_50_ values are reported for each cell line. Values are means ± standard deviation of 2 experiments in triplicate.

The analysis of the curves in Figure 6, shows that the compound **ZnPc-Sn_2_Ph_6_** is about 30 times more toxic on MCF-7 cells (LC_50_ = 0.016 µM) than the compound **ZnPc-SnPh_2_** (LC_50_ = 0.453 µM). It is also interesting to note that both tin dyads present a higher toxicity on cancer cells compared to healthy cells (Figure 6). Indeed, the LC_50_ are lower on cancer cells than on fibroblasts with LC_50_ of 0.016 vs. 0.032 µM for the compound **ZnPc-Sn_2_Ph_6_**, and LC_50_ of 0.453 and 0.513 for the compound **ZnPc-SnPh_2_**. These results suggest that the **ZnPc-SnPh_2_** dyad may be a potentially interesting basis to develop targeting cancer cells.

**Figure 7:**
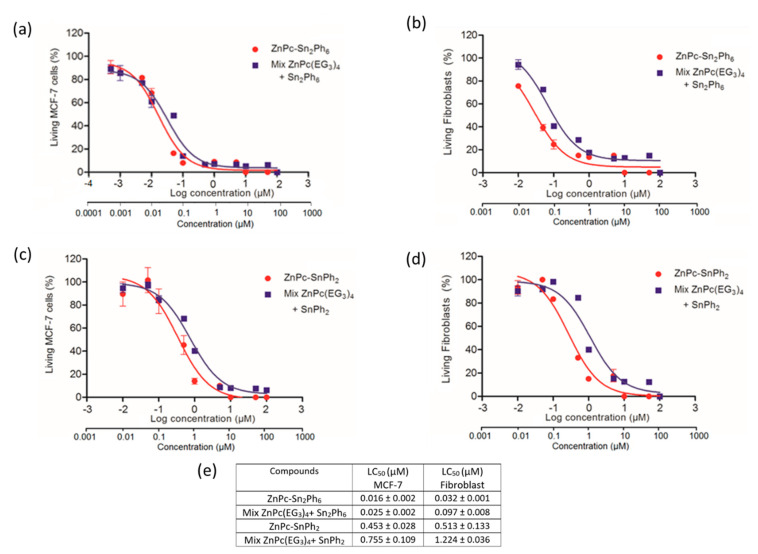
Study of the comparative cytotoxicity of the tin dyads with the equimolar mixture of their constituents, tin and zinc phthalocyanine. Comparison of the cytotoxic efficiency of the compound **ZnPc-Sn_2_Ph_6_** and the mixture **ZnPc(EG_3_)_4_** + **Sn_2_Ph_6_** (**a**) on MCF-7 cells and (**b**) on healthy fibroblasts. Comparison of the cytotoxic efficiency of the compound **ZnPc-SnPh_2_** and the mixture **ZnPc(EG_3_)_4_** + **SnPh_2_** (**c**) on MCF-7 cells and (**d**) on healthy fibroblasts. Cells were incubated during 72 h with increasing concentrations of the dyads or the mixture (Mix) and maintained in darkness. (**e**) LC_50_ values are reported for each cell line. Values are means ± standard deviation of 2 experiments in triplicate.

In order to confirm the therapeutic interest of using a dyad rather than its constituents, we compared the cytotoxicity of tin dyads to the cytotoxic effect of equimolar mixtures of the phthalocyanine **ZnPc(EG_3_)_4_** and **SnPh_2_** or **Sn_2_Ph_6_** (Figure 7). For all conditions tested here, and as reported in the Figure 7e, the cytotoxicity induced by dyads is always higher than the equimolar mixture of their constituents.

### 2.8. Photodynamic Therapy Investigations of Dyads

The PDT efficiency was first studied on cancer cells with the dyads **ZnPc-Sn_2_Ph_6_** and **ZnPc-SnPh_2_** under laser excitation of the zinc phthalocyanine in its Q-band at 650 nm. Experiments were performed at the concentration of LC_50_ and below up to a factor of 10. Several concentrations were tested, as reported in the tables below, (Table 4 and Table 5) but unfortunately no specific mortality due to the laser was obtained under these conditions. This result can be explained by fluorescence quenching of the zinc phthalocyanine in these dyads, which most probably inhibits the energy transfer process to oxygen.

Another series of experiments was carried out under light excitation 390/420 nm, on MCF-7 cells and also on healthy fibroblasts. Again, no effect could be observed.

Taken together, these results show that these two dyads have a cytotoxic effect on cells and would allow cancer cells to be targeted more effectively than healthy cells. However, they did not show efficacy in PDT under all of the conditions tested most probably because the singlet excited state is quenched by the appended tin complex, that inhibits singlet oxygen production.

## 3. Materials and Methods

### 3.1. Synthesis of the Compounds

#### Generalities

^1^H and ^13^C NMR spectra were recorded on an *AVANCE 300 UltraShield BRUKER* and *AVANCE 400 BRUKER.* Chemical shifts for ^1^H NMR spectra are referenced relative to residual proton in the deuterated solvent (CDCl_3_ δ = 7.26 ppm for ^1^H, DMSO-d_6_ δ = 2.50 ppm for ^1^H, THF-d_8_ δ = 3.57, 1.72 ppm for ^1^H) or to an internal reference (TMS, δ = 0 ppm for both ^1^H and ^13^C). NMR spectra were recorded at room temperature, chemical shifts are written in ppm and coupling constants in Hz. High-resolution mass (HR-MS) spectra were obtained by electrospray ionization coupled with high resolution ion trap orbitrap (LTQ-Orbitrap, ThermoFisher Scientific), working in ion-positive or ion-negative mode. UV-visible absorption spectra were recorded on a Variant Cary 300, using 1 cm path length cells. Emission spectra were recorded on a Fluoromax-4 Horiba Jobin Yvon spectrofluorometer (1 cm quartz cells).

Chemicals were purchased from Sigma-Aldrich or Alfa Aesar and used as received. CHCl_3_ is stabilized with EtOH. Thin-layer chromatography (TLC) was performed on silica sheets precoated with Merck 5735 Kieselgel 60F_254_. Column chromatography was carried out with Merck 5735 Kieselgel 60F (0.040–0.063 mm mesh). Reference **ZnPc(EG_3_)_4_** was synthesized according to literature [30].

**Phthalocyanine 3.** A mixture of **2** 66 mg (1.52 mmol), phthalonitrile **1** 3.1 g (10.68 mmol) and Zn(OAc)_2_.2H_2_O 83 mg (3.81 mmol) in n-pentanol (25 mL) was heated to 100°C, and a small amount of DBU (0.9 mL) was subsequently added. The mixture was heated at 150 °C for 12 h. After cooling the volatiles were removed under reduced pressure. The residue was dissolved in CHCl_3_ and a part of the symmetrical phthalocyanine was separated from the desired compound by flash chromatography using CHCl_3_/MeOH (98:2 *v*/*v*) as the eluent. The product was further purified by preparative TLC using the same eluent as before to give a blue green oily compound. Yield: (360 mg, 18%). UV-Vis (DMF, nm) λ_max_ (log Ꜫ/M^−1^ cm^−1^): 360 (4.83); 616 (4.46); 683 (5.19). ^1^H NMR (CDCl_3_, 300 MHz) δ (ppm): 9.12 (br, 4H), 8.68 (s, 4H), 7.61 (br, 4H), 4.66 (br, 8H), 4.16–4.06 (m, 12H), 3.91–3.90 (m, 6H), 3.84–3.83 (t, 2H, *J* = 4.95 Hz), 3.81–3.78 (m, 6H), 3.73–3.66 (m, 8H), 3.62–3.56 (m, 8H), 3.37 (s, 9H), 2.26–2.22 (t, 2H, *J* = 6.98 Hz), 1.45 (s, 3H), 1.24–1.18 (tt, 6H, *J* = 1.56, 7.34 Hz). HRMS (ES+) [M + H]^+^ m/z 1367.5042 found, 1367.5066 calc. Elem. Anal. Exp C, 58.97; H, 6.47; N, 7.74. calc C, 58.59; H, 6.1; N, 8.11.

**Phthalocyanine 4.** A mixture of **3** 70 mg (5 × 10^−2^ mmol), NaOH 18 mg (45 × 10^−2^ mmol), and acetone (8 mL) was heated under reflux for 2 h. The volatiles were removed under reduced pressure. The green residue was washed thoroughly with acetone, and then redissolved in water and acidified with 3 M HCl until pH = 4. The green precipitate was washed thoroughly with water and ethanol, and then dried under vacuum. Yield: (57 mg, 85%). UV-Vis (DMF, nm) λ_max_ (log Ꜫ/M^−1^ cm^−1^): 360 (4.86); 616 (4.48); 683 (5.21). ^1^H NMR (DMSO-d_6_ with a trace amount of pyridine, 300 MHz) δ (ppm): 9.29–9.24 (m, 4H), 8.93–8.88 (m, 4H), 7.79–7.75 (m, 4H), 4.68–4.63 (br, 8H), 4.07–4.04 (br, 8H), 3.64–3.66 (m, 10H), 3.57–3.52 (m, 12H), 3.42–3.40 (t, 8H, *J* = 6.06 Hz), 3.23 (s, 9H), 2.23–2.20 (2H, m), 1.36 (s, 3H). HRMS (ES-) [M-H] m/z 1309.4309 found, 1309.4238 calc. Elem. Anal. Exp C, 54.4; H, 5.52; N, 7.95. calc C, 54.82; H, 5.95; N, 8.12.

**ZnPc-SnPh_2_.** A solution of phthalocyanine **4** 35 mg (2.6 × 10^−2^ mmol) in THF (1 mL) was added dropwise to a solution of SnPh_2_Cl_2_ 9 mg (2.6 × 10^−2^ mmol) in THF (1 mL) at room temperature. The reaction was stirred for 20 min, subsequently NEt_3_ 8 µL (5.3 × 10^−2^ mmol) was added dropwise. The reaction was then stirred overnight. The solvent was evaporated and the residue was washed with water and dried in vacuum. Yield: (42 mg, 100%). UV-Vis (DMF, nm) λ_max_ (log Ꜫ/M^−1^ cm^−1^): 360 (4.82); 616 (4.45); 683 (5.17). ^1^H NMR (THF-d_8_, 400 MHz) δ (ppm): 9.26–9.24 (br, 4H), 8.90 (br, 4H), 7.77–7.72 (m, 8H), 7.45–7.37 (m, 6H), 4.68–4.66 (t, 8H, *J* = 4.71 Hz), 4.12–4.10 (t, 8H, *J* = 4.48 Hz), 3.84–3.82 (t, 8H, *J* = 5.17 Hz), 3.75–3.71 (m, 8H), 3.67–3.64 (t, 8H, *J* = 4.82 Hz), 3.52–3.50 (m, 8H), 3.31 (s, 9H), 1.28 (s, 3H). HRMS (ES+) [M + H]^+^ m/z 1583.4023 found, 1583.4088 calc. Elem. Anal. Exp C, 51.43; H, 5.82; N, 6.63. calc C, 51.63; H, 5.77; N, 6.42.

**ZnPc-Sn_2_Ph_6_.** A solution of phthalocyanine **4** 33 mg (2.5 × 10^−2^ mmol) in THF (1 mL) was added dropwise to a solution of SnPh_3_Cl 19 mg (5 × 10^−2^ mmol) in THF (1 mL) at room temperature. The reaction was stirred for 20 min, subsequently NEt_3_ 7 µL (5 × 10^−2^ mmol) was added dropwise. The reaction was then stirred overnight. The solvent was evaporated and the residue was washed with water and dried in vacuum. Yield: (50 mg, 100%). UV-Vis (DMF, nm) λ_max_ (log Ꜫ/M^−1^ cm^−1^): 360 (4.84); 616 (4.47); 683 (5.2). ^1^H NMR (DMSO-d_6_ with a trace amount of pyridine, 300 MHz) δ (ppm): 9.05–9.04 (br, 4H), 8.66–8.61 (br, 4H), 7.85–7.82 (br, 6H), 7.71–7.64 (br, 6H), 7.43–7.41 (br, 9H), 7.28 (br, 13H), 4.64–4.62 (br, 8H), 4.08 (br, 8H), 3.82–3.79 (br, 6H), 3.71–3.69 (br, 8H), 3.63–3.62 (br, 8H), 3.51–3.48 (t, 8H, *J* = 4.88 Hz), 3.26 (s, 9H), 1.19 (s, 3H). HRMS (ES+) [M + H]^+^ m/z 2011.4662 found, 2011.4675 calc. Elem. Anal. Exp C, 57.28; H, 6.12; N, 5.54. calc C, 57.37; H, 5.88; N, 5.78.

### 3.2. Biological Experiments

#### 3.2.1. Cell Culture

Human breast adenocarcinoma cells (MCF-7) (purchased from ATCC) were cultured in Dulbecco Eagle’s Minimal Essential Medium (DMEM) supplemented with 10% fetal bovine serum (FBS) and 1% penicillin/streptomycin (P/S). Adult Human Dermal Fibroblast cells (FS 20-68) were maintained in Roswell Park Memorial Institute (RPMI) medium supplemented with 10% FBS and 1% P/S. Both cell lines were allowed to grow in humidified atmosphere at 37 °C under 5% CO_2_. For cytotoxic studies, the compounds (powder) were first diluted in DMSO at the concentration of 10 mM. Then, they were sonicated during 30 s and diluted at the required concentrations in culture medium of each cell line.

#### 3.2.2. Cytotoxicity Study

MCF-7 and fibroblasts (FS 20-68) cells were seeded into 96-well plates at a density of 1000 cells cm^−2^. One day after cell growth, cells were incubated with or without different concentrations of compounds (from 0.01 to 100 μM) for 3 days. To quantify the percentage of living cells in each condition, cells were incubated 4 h with 0.5 mg.mL^−1^ MTT (3-(4, 5-dimethylthiazol-2-yl)-2,5-diphenyltetrazoliumbromide) in order to determine mitochondrial enzyme activity. Then, MTT precipitates were dissolved in ethanol/DMSO (1:1) solution and absorbance was measured at 540 nm.

#### 3.2.3. Phototoxicity Assay at 650 nm

MCF-7 cancer cells were seeded into 96-well plates at a concentration of 1000 cells per well in 100 µL of culture medium and allowed to grow for 24 h. Then, cells were incubated 24 h, with or without various concentrations of compound solutions. After incubation, cells were submitted or not to laser irradiation (650 nm; 39 J·cm^–2^) during 20 min. Two days after irradiation, MTT assay was performed to measure the level of living cells.

### 3.3. Statistical Analysis

Statistical analysis was performed using the Student’s *t*-test to compare paired groups of data. A *p* value < 0.05 was considered as statistically significant.

### 3.4. Singlet Oxygen Quantum Yield

Absorption spectra were recorded on a UV-3600 UV-visible double beam spectrophotometer (SHIMADZU, Marne La Vallee, France). Fluorescence and singlet oxygen spectra were recorded on a Fluorolog FL3-222 spectrofluorimeter (HORIBA Jobin Yvon, LONGJUMEAU, Paris, France) equipped with 450 W Xenon lamp, a thermo-stated cell compartment (25 °C), a UV-visible photomultiplier R928 (Hamamatsu, Japan) and an InGaAs infrared detector (DSS-16A020L Electro-Optical System Inc., Phoenixville, PA, USA).

Excitation beam is diffracted by a double ruled grating SPEX monochromator (1200 grooves/mm blazed at 330 nm). Emission beam is diffracted by a double ruled grating SPEX monochromator (1200 grooves/mm blazed at 500 nm). Singlet oxygen emission was detected through a double ruled grating SPEX monochromator (600 grooves/mm blazed at 1 µm) and a long-wave pass (780 nm). All spectra were measured in 4 faces quartz cuves. All the emission spectra (fluorescence and singlet oxygen luminescence) have been displayed with the same absorbance (less than 0.2) with the lamp and photomultiplier correction. The standard used for the singlet oxygen quantum yield was the Zinc(II) 2,9,16,23-(tetra-t-butyl)phthalocyanine with a standard quantum yield of 0.25.

## 4. Conclusions

Two new dyads **ZnPc-SnPh_2_**, **ZnPc-Sn_2_Ph_6_** were prepared, by combining a zinc phthalocyanine sensitizer with a cytotoxic entity such as a tin-based complex. Cytotoxicity studies of the **ZnPc-SnPh_2_** and **ZnPc-Sn_2_Ph_6_** dyads on cancer cells (MCF-7) and on healthy fibroblasts show that they exhibit high cytotoxicity. In particular, the dyad **ZnPc-Sn_2_Ph_6_** containing two tin atoms is highly potent towards cancer cells (LC_50_ = 0.16 μM) and is twice less toxic towards healthy human fibroblasts. In contrast, no PDT activity was detected for these two dyads at concentrations close to and below LC_50_, most probably due to quenching of the singlet excited state of phthalocyanine by the tin complex, which decreased the singlet oxygen quantum yield. This result highlights that for the development of conjugates consisting of a sensitizer connected to a chemotherapeutic agent, one must pay attention that the photophysical properties of the sensitizer and in particular its singlet oxygen quantum yield is maintained in the conjugate in order to combine the PDT activity with the cytotoxicity of the anticancer drug. This can be estimated from Stern-Volmer quenching experiments before synthesizing the conjugates. This aspect has been mostly overlooked in many previous studies dealing with similar approach.

## Figures and Tables

**Table 1:** Maximum absorption wavelengths and molar extinction coefficients of the compounds.

Compounds	λ_abs_/nm (log Ꜫ/M^−1^ cm^−1^)
**3 ^a^**	360 (4.83); 616 (4.46); 683 (5.19)
**4 ^a^**	360 (4.86); 616 (4.48); 683 (5.21)
**ZnPc(EG_3_)_4_^b^**	351 (5.45); 615 (5.04); 681 (5.71)
**ZnPc-SnPh_2_^a^**	360 (4.82); 616 (4.45); 683 (5.17)
**ZnPc-Sn_2_Ph_6_^a^**	360 (4.84); 616 (4.47); 683 (5.2)

^a^ recorded in DMF at room temperature. ^b^ recorded in CHCl_3_ at room temperature.

**Table 2:** Wavenumber values of the stretching bands of the compounds recorded in the solid state by IR-ATR spectroscopy.

Compound	ν(Sn-O) cm^−1^	ν_as_(OCO) cm^−1^	ν_s_ (OCO) cm^−1^	ν
**4**	-	1725	1396	329
**ZnPc-SnPh_2_**	452	1557	1430sh	127
**ZnPc-Sn_2_Ph_6_**	454	1547	1429	118

**Table 3:** Singlet oxygen quantum yields of the compounds determined in CHCl_3_ and D_2_O + 1% of DMSO upon excitation at 615 nm.

Compound	In CHCl_3_	In D_2_O + 1% of DMSO
**ZnPc(EG_3_)_4_**	0.21 ± 0.05	0 ± 0.05
**ZnPc-SnPh_2_**	0.09 ± 0.05	0 ± 0.05
**ZnPc-Sn_2_Ph_6_**	0.12 ± 0.05	0 ± 0.05

**Table 4:** Values of cellular survival in % corresponding to the mean ± standard deviation of 2 independent experiments conducted in duplicate.

MCF-7 with ZnPc-Sn_2_Ph_6_ (in µM)	0	0.005	0.01	0.05
% cell survival, in dark	100 ± 2	99 ± 3	77 ± 2	38 ± 3
% cell survival, λ_exc_ = 650 nm	98 ± 1	98 ± 4	75 ± 3	41 ± 3

**Table 5:** Values of cellular survival in % corresponding to the mean ± standard deviation of 2 independent experiments conducted in duplicate.

MCF-7 with ZnPc-SnPh_2_ (in µM)	0	0.05	0.1	0.5
% cell survival, in dark	100 ± 5	92 ± 3	88 ± 4	38 ± 5
% cell survival, λ_exc_ = 650 nm	90 ± 9	90 ± 4	80 ± 2	36 ± 2

## Data Availability

Raw data is available from the corresponding author upon request.

## Supplementary Materials

The following are available online at https://www.mdpi.com/article/10.3390/ph14050413/s1, Figure S1: UV-Vis. absorption spectra of compounds **SnPh_2_** and **Sn_2_Ph_6_** recorded in dichloromethane.; Figure S2: Mass spectrum of phthalocyanine **3**; Figure S3: Mass spectrum of phthalocyanine **4**; Figure S4: Mass spectrum of compound **ZnPcSnPh_2_**; Figure S5: Mass spectrum of compound **ZnPcSn_2_Ph_6_**; Figure S6: ^1^H NMR spectrum of phthalocyanine **3** recorded in CDCl_3_; Figure S7: ^1^H NMR spectrum of phthalocyanine **4** recorded in DMSO-d_6_ with a trace of pyridine; Figure S8: ^1^H NMR spectrum of compound **ZnPc-SnPh_2_** recorded in THF-d_8_; Figure S9: ^1^H NMR spectrum of compound **ZnPc-Sn_2_Ph_6_** recorded in DMSO-d_6_ with a trace of pyridine.
