# Supplementary material for: Study of Cytotoxic and Photodynamic Activities of Dyads Composed of a Zinc Phthalocyanine Appended to an Organotin
Source: Pharmaceuticals (Basel). 2021 Apr 28;14(5):413. doi: 10.3390/ph14050413 (PMC8145453; doi:10.3390/ph14050413)
Supplement: Supplementary file 1 [file pharmaceuticals-14-00413-s001.zip › pharmaceuticals-1193521-supplementary.pdf]

Supporting information for

Study of cytotoxic and photodynamic activities of dyads composed of a zinc phthalocyanine appended to an organotin

Isabelle Toubia,[ac#] Christophe Nguyen,[b#] Stéphane Diring,[a] Marine Pays,[b] Elodie Mattana,[b] Philippe Arnoux[c], Céline Frochot,*[c] Magali Gary-Bobo,*[b] Marwan Kobeissi,*[d] Fabrice Odobel*[a]

[a]*CEISAM, Chimie Et Interdisciplinarité, Synthèse, Analyse, Modélisation, CNRS, UMR CNRS 6230, UFR des Sciences et des Techniques ; 2, rue de la Houssinière - BP 92208; 44322 NANTES Cedex 3 (France). E-mail:Fabrice.Odobel@univ-nantes.fr*

[b]*IBMM, Univ Montpellier, CNRS, ENSCM, Montpellier, France. E-mail : magali.gary-bobo@inserm.fr*

[c]*LRGP, Laboratoire Réactions et Génie des Procédés, UMR 7274 CNRS-Université de Lorraine, 1 rue Grandville, 54000 Nancy, France. E-mail : celine.frochot@univ-lorraine.fr*

[d]Laboratoire RammalRammal, Equipe de Synthèse Organique Appliquée SOA, Université Libanaise, Faculté des Sciences 5, Nabatieh, Liban. *E-mail: mkobeissi@ul.edu.lb*

[#]These authors contributed equally to this study.

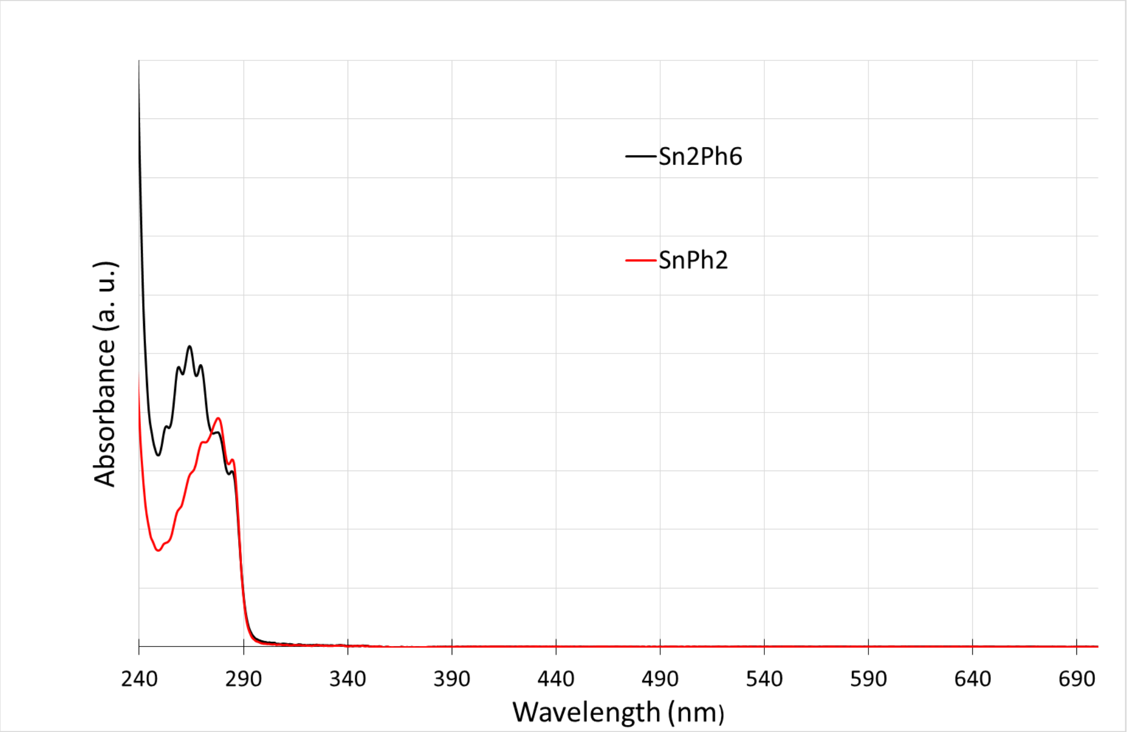

Figure S1. Overlay of the UV-Vis. absorption spectra of compounds **$SnPh_2$** and **Sn_2Ph_6** recorded in dichloromethane.

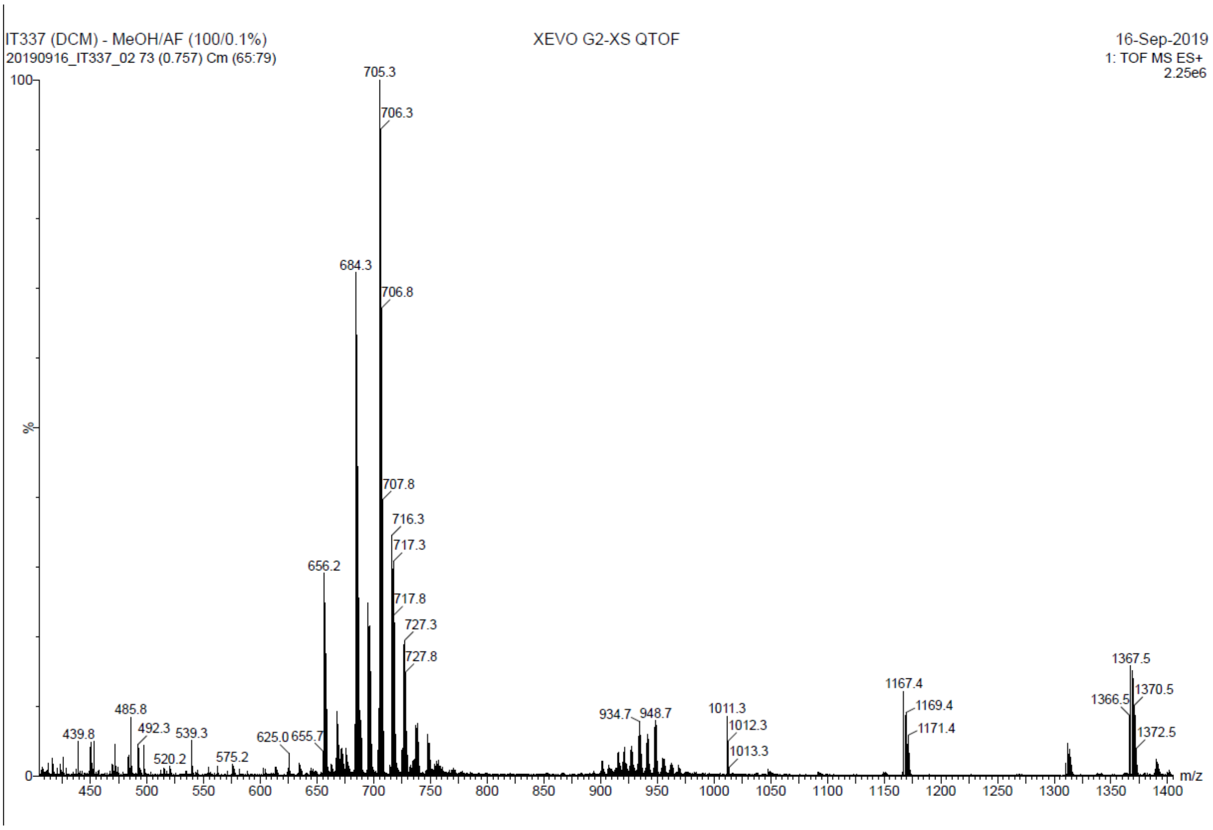

Figure S2. Mass spectrum of phthalocyanine **3**.

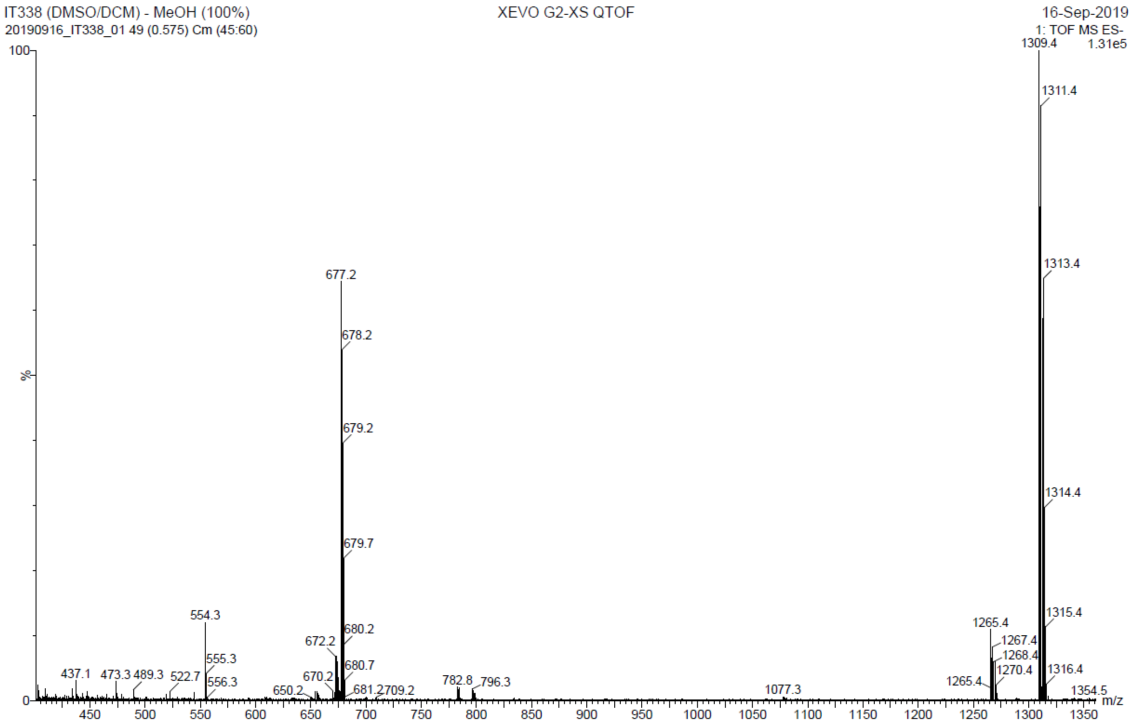

Figure S3. Mass spectrum of phthalocyanine **4**.

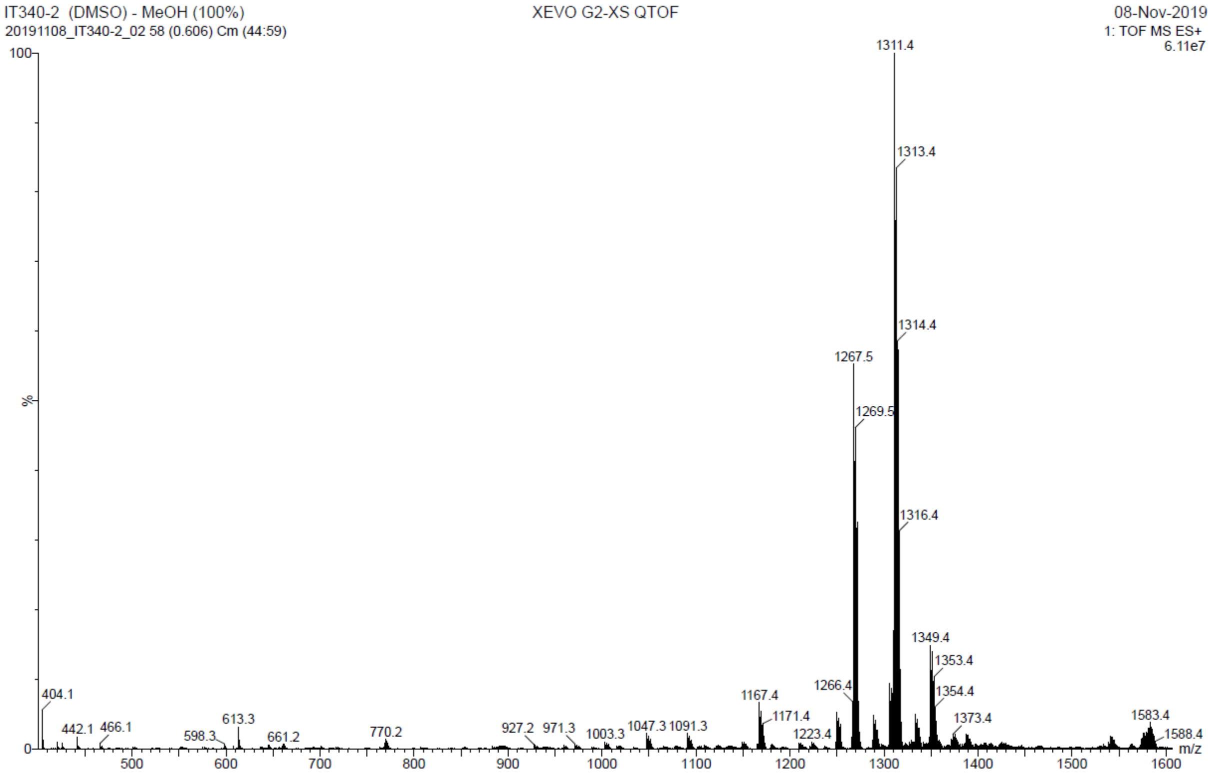

Figure S4. Mass spectrum of compound **ZnPcSnPh$_2$**

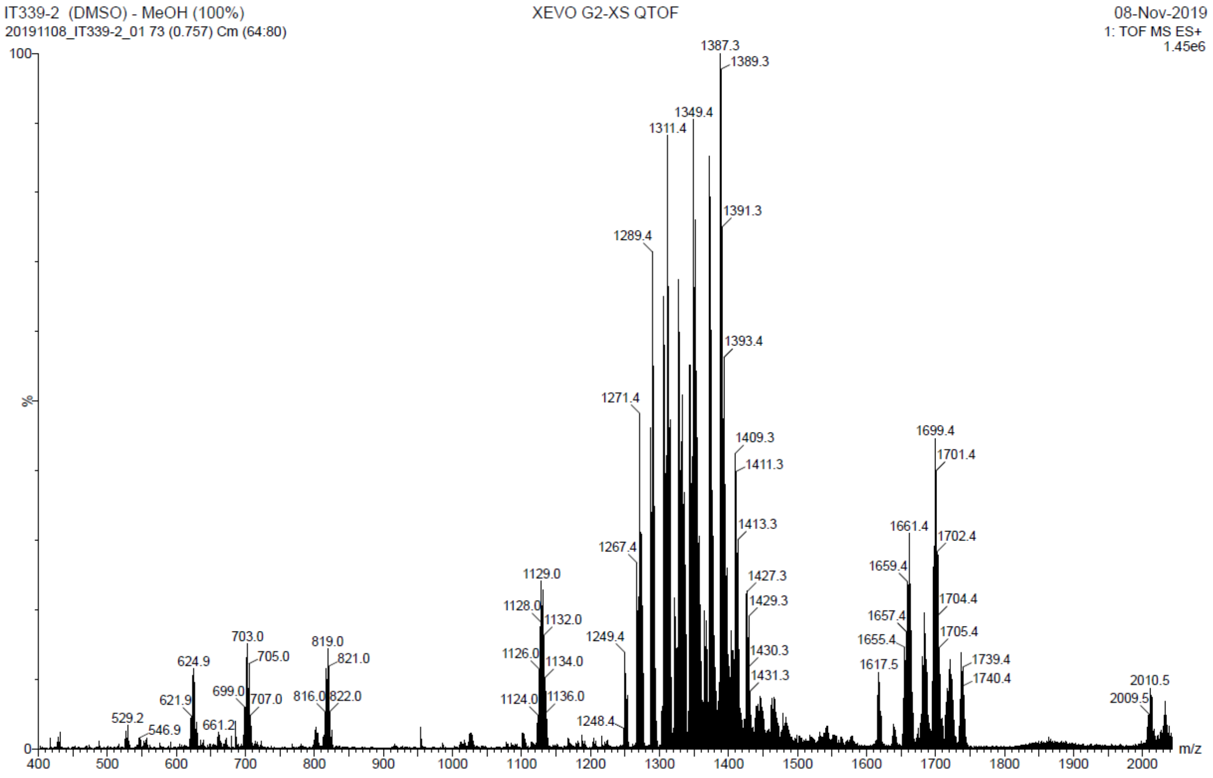

Figure S5. Mass spectrum of compound **ZnPcSn$_2$Ph$_6$**

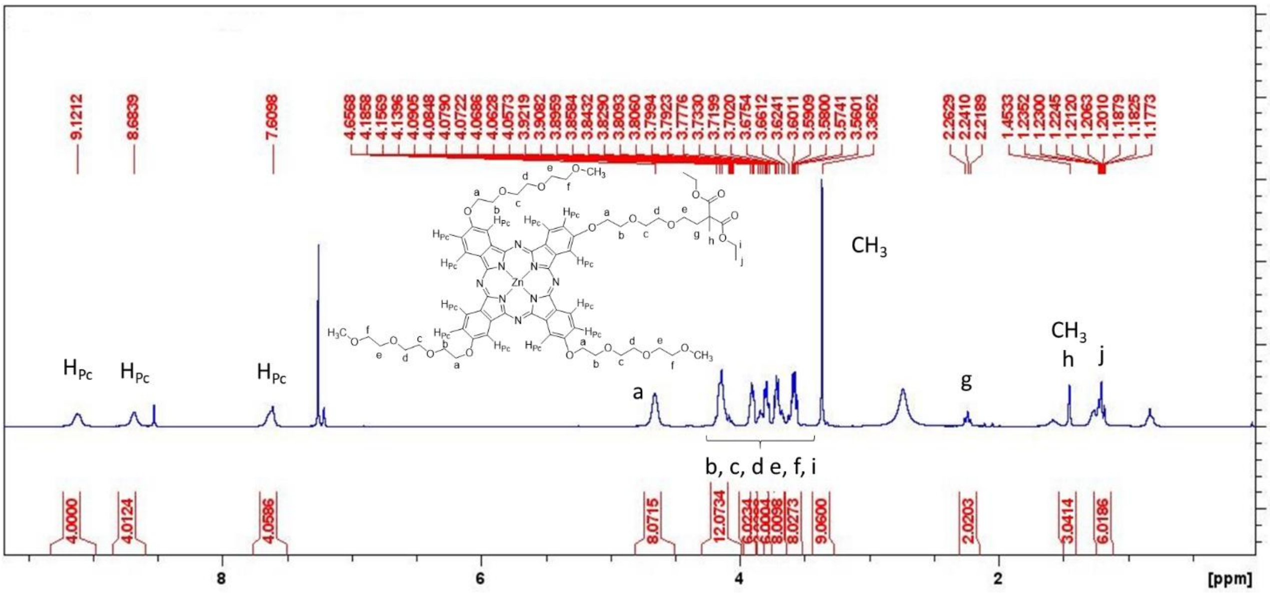

Figure S6. ^{1}H NMR spectrum of phthalocyanine **3** recorded in $CDCl_3$.

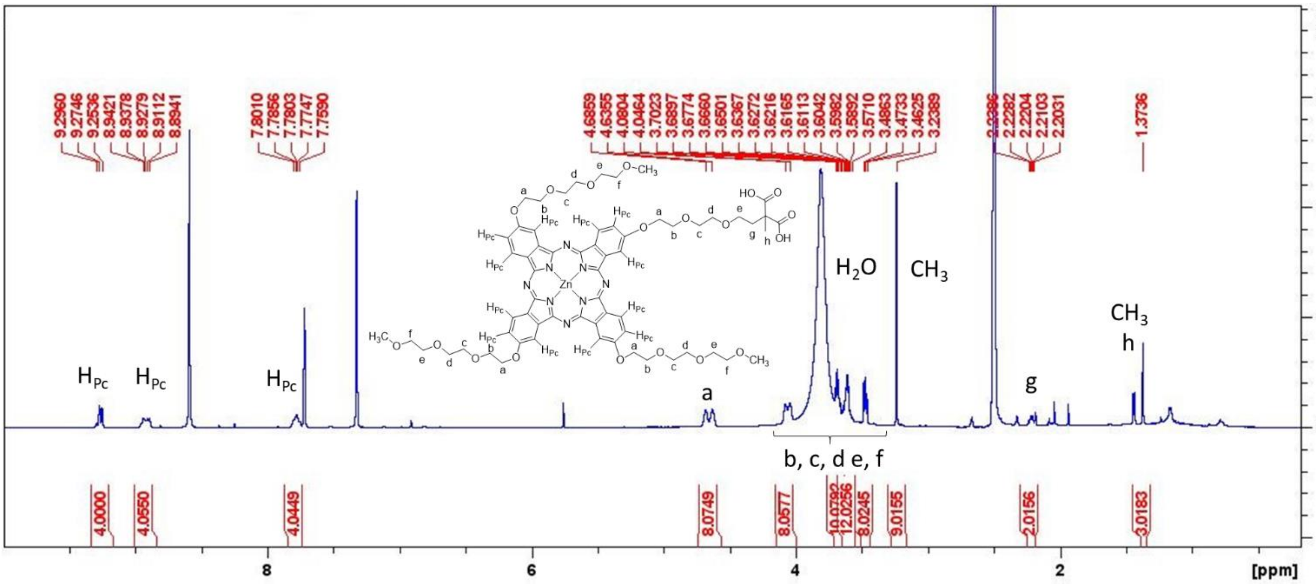

Figure S7. ^{1}H NMR spectrum of phthalocyanine **4** recorded in DMSO-d_6 with a trace of pyridine.

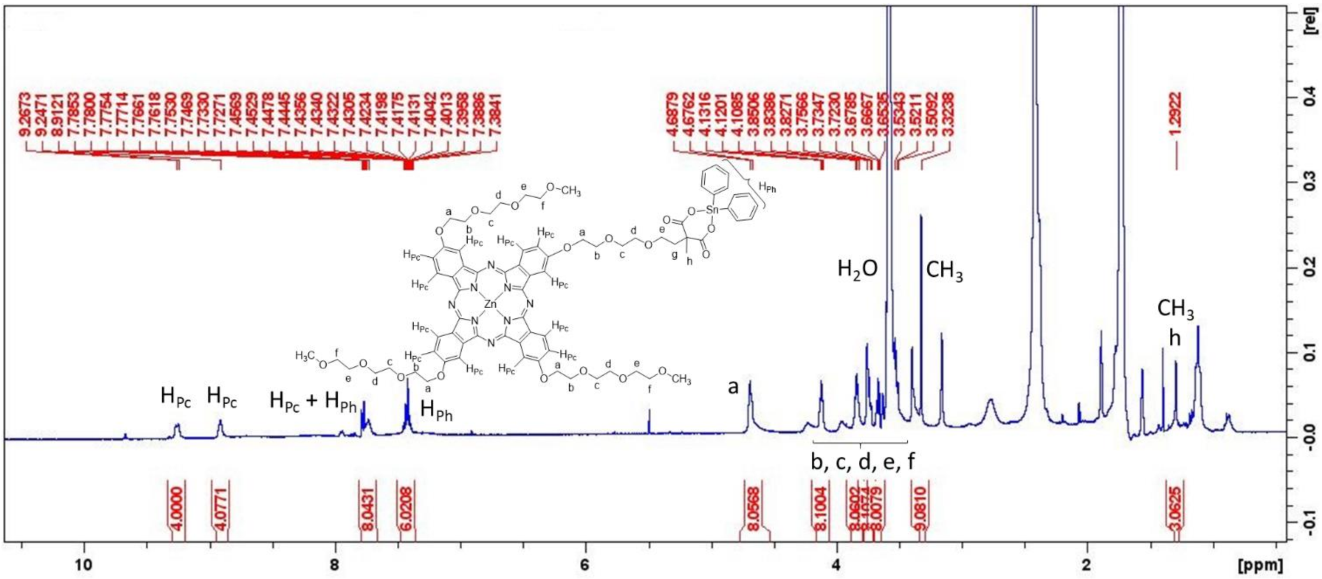

Figure S8. ^{1}H NMR spectrum of compound **ZnPc-SnPh$_2$** recorded in THF-d$_8$.

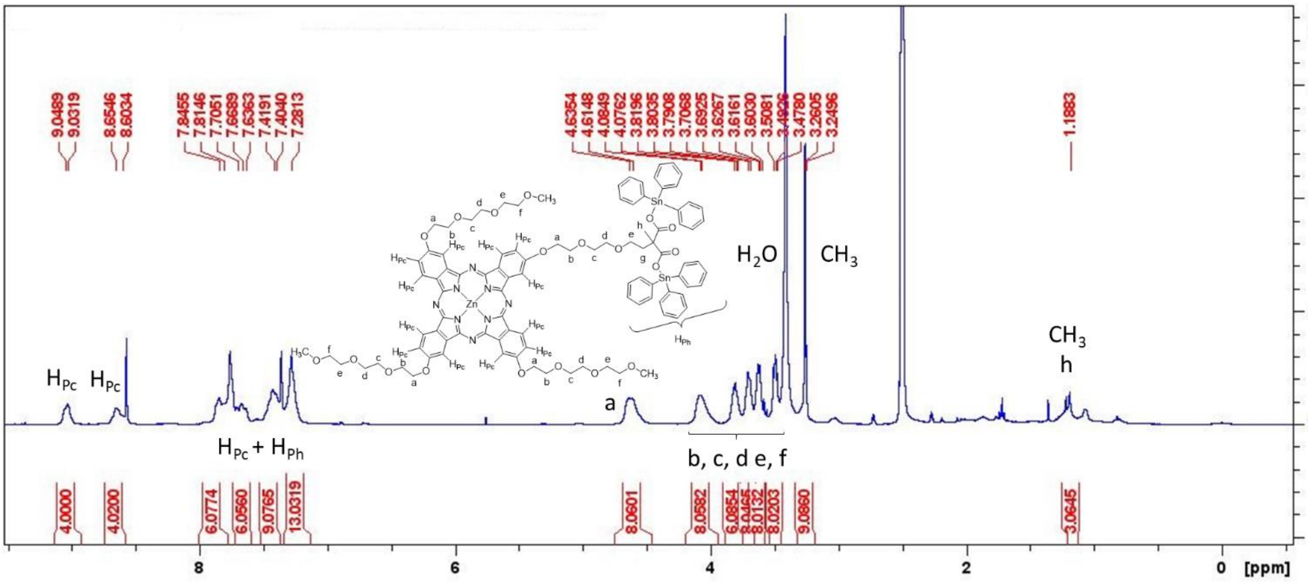

Figure S9. ^{1}H NMR spectrum of compound **ZnPc-Sn$_2$Ph$_6$** recorded in DMSO-d$_6$ with a trace of pyridine.
